# Health disparities between lesbian, gay, and bisexual adults and the general population in South Korea: Rainbow Connection Project I

**DOI:** 10.4178/epih.e2017046

**Published:** 2017-10-19

**Authors:** Horim Yi, Hyemin Lee, Jooyoung Park, Bokyoung Choi, Seung-Sup Kim

**Affiliations:** 1 Department of Public Health Sciences, Graduate School of Korea University, Seoul, Korea; 2 Department of Social and Behavioral Sciences, Harvard T. H. Chan School of Public Health, Boston, MA, USA

**Keywords:** Sexual minorities, Health status disparities, Minority health, Republic of Korea

## Abstract

**OBJECTIVES:**

This study aims to investigate health disparities between lesbian, gay, and bisexual (LGB) adults and the general population in Korea, where there is low public acceptance of sexual minorities and a lack of research on the health of sexual minorities.

**METHODS:**

The research team conducted a nationwide survey of 2,335 Korean LGB adults in 2016. Using the dataset, we estimated the age-standardized prevalence ratios (SPRs) for poor self-rated health, musculoskeletal pain, depressive symptoms, suicidal behaviors, smoking, and hazardous drinking. We then compared the SPRs of the LGB adults and the general population which participated in three different nationally representative surveys in Korea. SPRs were estimated for each of the four groups (i.e., gay men, bisexual men, lesbians, and bisexual women).

**RESULTS:**

Korean LGB adults exhibited a statistically significantly higher prevalence of depressive symptoms, suicidal ideation and attempts, and musculoskeletal pain than the general population. Lesbian and bisexual women had a higher risk of poor self-rated health and smoking than the general women population, whereas gay and bisexual men showed no differences with the general men population. Higher prevalence of hazardous drinking was observed among lesbians, gay men, and bisexual women compared to the general population, but was not observed in bisexual men.

**CONCLUSIONS:**

The findings suggest that LGB adults have poorer health conditions compared to the general population in Korea. These results suggest that interventions are needed to address the health disparities of Korean LGB adults.

## INTRODUCTION

Previous literatures have well-documented the health disparities experienced by the sexual minorities. These studies have reported that compared to the general population, lesbian, gay, and bisexual (LGB) individuals are having more adverse health conditions [1-11]. LGB people have poorer mental health statuses [3-5, 10,11], with more prevalent suicidal ideation [6]. Similarly, the prevalence of several chronic diseases [3], including insomnia and dermatitis [1], is higher in the LGB population than in the general population. Furthermore, higher prevalence of risky health behavior is observed among LGB adults, such as heavy drinking [8,9] or smoking [2,8,9].

Despite such findings, studies on the health of LGB people are lacking in South Korea (hereafter Korea). According to a systematic review by Lee et al. [12], there are only 123 published studies that have investigated the health of sexual minorities in Korea as of 2013. Among them, 101 articles (78.9%) were clinical investigations, mostly case reports of sex reassignment surgeries of transgender and intersex individuals by healthcare institutions. Only 30 out of the 123 studies have examined the health status of LGB individuals, with even a fewer number of studies (n= 25) analyzing the health disparities and the social factors that may be associated with their health.

Due to the paucity of existing studies in the field, previous literatures regarding health disparities of LGB population in Korea have the following limitations. First, most studies were on the health disparities of LGB adolescents, using the Korea Youth Risk Behavior Web-based Survey which is the only nationally representative data that includes sexual orientation in the questionnaire [13-15]. On the other hand, because of the absence of sexual orientation measure in national health surveys targeting adults, there are only few studies on health disparities of adult sexual minorities. Second, no previous research has compared the health status of lesbian and bisexual individuals with the general population. There were few studies examining health disparities of gay men or men who have sex with men (MSM) [16-19], but there was no study on the health disparities of lesbian and bisexual women. Also, studies on MSM have not distinguished between homosexual and bisexual men [16]. Third, studies compared the health status of gay men and MSM with heterosexual adult men sampled these populations separately for their analysis [16-19] and sample size of those studies was relatively small with the largest such study only having 129 MSM participants. Lastly, the health outcomes investigated in the existing studies are limited to only some mental health conditions and health behaviors. Despite previous studies reporting that the Korean LGB population has a higher prevalence of risky health behaviors [14], and have higher prevalence of depressive symptoms [14,17,20] and suicide risk [15,17], none of these studies have studied physical health.

To fill these gaps in knowledge, this study aimed to explore the health disparities between LGB adults and the general population in Korea. The purpose of this study was to compare the health status of LGB individuals and the general population in diverse aspects, including self-rated health, mental health (depressive symptoms, suicidal ideation, suicide attempts), physical health (musculoskeletal pains), and health-related behaviors (smoking and hazardous drinking).

## MATERIALS AND METHODS

### Study population

The data were collected as part of “Rainbow Connection Project I – Korean Lesbian, Gay, & Bisexual Adults’ Health Study.” Participants were eligible if they were aged 19 years or older, living in Korea, and self-identified as LGB.

The survey was administered online for four weeks, from November 11 to December 5, 2016. Participants were recruited by posting ads on two websites frequented by Korean sexual minorities and through the social network services (Facebook and Twitter). This study protocol was approved by the institutional review board (IRB) of Korea University (IRB no. 1040548-KU-IRB-16191-A-1), and informed consent for participation and academic use of data were collected from all participants. All participants were rewarded 5,000 Korean won as compensation for participation.

A total of 2,430 people responded to the survey, and we excluded 17 people who did not consent to study participation or academic use of data and 72 people for not providing socio-demographic information. Further, to prevent bias caused by a heterogeneity of age distribution between the LGB and general population, five men in the 61-69 years age range and one woman in the 51-69 years age range were excluded, resulting in a total of 2,335 participants included in the final analysis.

### Measurements

The survey comprised of 173 items in four categories: socio-demographic, sexual orientation, social experience, and health status.

Self-rated health was rated on a 5-point scale as “very good,” “good,” “fair,” “poor,” and “very poor.” Responses indicating “poor” and “very poor” were classified into the poor self-rated health group. For physical health status, nine physical symptoms were investigated, and musculoskeletal pains were included in this study. The respondents answered “yes” or “no” to three items about having a) back pain, b) muscle pain in the upper limbs, and c) muscle pain in the lower limbs over the past 12 months. Results on six other physical symptoms are shown in Appendix 1.

Although depressive symptoms were measured using the Center for Epidemiologic Studies-Depression Scale-D (CES-D) 20 in the survey, the CES-D 11 was used because the Korea Welfare Panel Study (KWPS), which is a dataset used for the comparison, measured depressive symptoms with CES-D 11. Each item on the CES-D 11 is rated on a 4-point scale (0-3 points), with a maximum score of 33 points. The total score was calculated to a maximum score of 60 points by multiplying 20/11, and a final score of 16 or greater was classified as having depressive symptoms. Suicidal ideation was measured by the question “Have you ever had a serious thought about dying by suicide over the past 12 months?” and suicide attempts were measured by the question “Have you ever attempted to die by suicide over the past 12 months?”.

Smoking was measured by asking about pack-year and current smoking status. People who responded to have smoked “more than five packs (100 cigarettes) during their lifetime” and to smoke currently were classified as smokers. Hazardous drinking was measured based on the drinking frequency and volume. Following the Korean national guideline of the hazardous drinking [21], people who drink more than 7 drinks (for men) and 5 drinks (for women) per occasion and more than two time per week were classified as hazardous drinkers.

### Statistical analysis

This study assessed the health status of the Korean LGB adults and compared them with those of the general population. To assess prevalence of health outcomes among the general population in Korea, we analyzed several nationally representative surveys with the closet study period. Those surveys had the same measures to assess health outcomes which were used in the survey of Rainbow Connection Project I. More specifically, the Korea National Health and Nutrition Examination Survey VI (KNHANES VI) was used for self-rated health, suicidal ideation and suicide attempts over the past 12 months, and the 4th Korean Working Condition Survey (4th KWCS) was used for musculoskeletal pains. The 10th KWPS was used for depressive symptoms, and the 3rd KWCS was used for smoking and hazardous drinking.

To compare the health status of LGB adults and the general population, indirect standardization was performed, considering heterogeneity of age distribution between the general population in the nationally representative surveys and the LGB adults in the survey of Rainbow Connection Project I. For each health conditions, we calculated standardized prevalence ratio (SPR) as the ratio of the observed number of cases among LGB adults compared with the expected number of cases based on age-specific prevalence derived from the general population participated in nationally representative surveys, and their 95% confidence intervals (CIs). Men aged 61 or higher and women aged 51 or higher were excluded in the data analysis because there was a small number of LGB participants above those ages. To ensure representativeness of the population, age-specific prevalence of the general population was calculated by weighting each data with cross-sectional individual weights using commands svyset and svy on Stata. SPRs were estimated for each of the four sexual identity groups separately (i.e., gay, bisexual men, lesbian, bisexual women). SPRs for gay and bisexual men and SPRs for lesbians and bisexual women were calculated with men and women in the general population, respectively. All statistical analyses were performed using the Stata/SE version 13.0 (StataCorp., College Station, TX, USA).

## RESULTS

### Socio-demographic characteristics

The participants’ demographic characteristics are shown in Table 1. Among total participants, 39.1% (n= 913) were gay men, 5.2% (n= 122) were bisexual men, 26.5% (n= 619) were lesbians, and 29.2% (n= 681) were bisexual women, showing a relatively low participation from bisexual men compared to the other three groups. A total of 90.9% (n= 2,122) of the respondents were between the ages 19-39, while 9.1% (n=213) were older than 40 years. The majority of the participants (68.1%, n= 1,591) were currently attending, or graduated from a four-year college, followed by those currently attending, or graduated from a community college (12.1%, n= 282), graduate school (11.3%, n= 263), and participants with school graduation or lower (8.5%, n= 199). The greatest number of participants had an annual household income higher than 50 million Korean won (22.2%, n= 516). In terms of employment status, 33.5% (n= 777) of the respondents were currently students, followed by regular full-time workers (25.4%, n= 590), non-regular workers including fixed-term, hourly, contract, and special employment (24.4%, n= 566), unemployed (11.2%, n= 259), selfemployed or employer (4.6%, n = 106), and non-paid family worker (1.0%, n= 24). The greatest number of participants lived in Seoul (47.8%, n= 1,113), followed by other metropolitan cities (19.5%, n= 453), and other regions (32.7%, n= 762).

### Comparison of the health status of Korean adult lesbian, gay, and bisexual and general populations

Table 2 shows that 12.2% (n= 111) of gay men, 7.6% (n= 9) of bisexual men, 22.3% (n= 138) of lesbians, and 27.1% (n= 184) of bisexual women rated their health to be “poor” or “very poor.”

In order to compare the prevalence of poor self-rated health in the Korean LGB adults with that in the general population, SPRs were calculated using data from 4,546 men (age, 19-60 years) and 4,432 women (age, 19-50 years) who participated in the KNHANES VI (2013-2015) as a general population. When compared to women in the general population, lesbians (SPR, 1.80; 95% CI, 1.51 to 2.13) and bisexual women (SPR, 2.24; 95% CI, 1.93 to 2.59) reported poor self-rated health 1.80 times and 2.24 times more frequently, respectively. However, there were no statistically significant differences in the self-rated health between gay and bisexual men and men in the general population (Table 2).

SPRs of musculoskeletal pains over the past 12 months were calculated using data from 20,075 economically active men and 14,066 economically active women who participated in the 4th KWCS (2014) as a general population. The prevalence of back pain, upper limbs pain, and lower limbs pain was statistically significantly higher among LGB adults compared to the general population. For example, the prevalence of back pain was 2.85-3.40 times higher among gay men (SPR, 2.85; 95% CI, 2.48 to 3.25) and bisexual men (SPR, 3.40; 95% CI, 2.29 to 4.85). Further, the prevalence of back pain was 3.88-5.39 times higher among lesbians (SPR, 3.88; 95% CI, 3.41 to 4.41) and bisexual women (SPR, 5.39; 95% CI, 4.83 to 6.01) (Table 3).

Table 4 shows that 33.9% (n= 305) of gay men, 36.4% (n= 43) of bisexual men, 47.2% (n= 288) of lesbians, and 59.2% (n= 398) of bisexual women experienced depressive symptoms.

SPRs for depressive symptoms were calculated using data from 3,308 men and 2,705 women who participated in the 10th KWPS (2015). Compared to the general population, the prevalence of depressive symptoms was 7.13-7.16 times higher among gay men (SPR, 7.13; 95% CI, 6.36 to 7.98) and bisexual men (SPR, 7.16; 95% CI, 5.18 to 9.65), and 5.08-6.12 times higher among lesbians (SPR, 5.08; 95% CI, 4.51 to 5.70) and bisexual women (SPR, 6.12; 95% CI, 5.54 to 6.76) (Table 4).

To the question “Have you ever had a serious thought about dying by suicide in the past 12 months?”, 25.7% (n= 233) of gay men, 28.7% (n= 35) of bisexual men, 34.6% (n= 214) of lesbians, and 47.6% (n= 323) of bisexual women answered “yes.” To the question “Have you ever attempted to die by suicide in the past 12 months?”, 3.6% (n= 33) gay men, 4.2% (n= 5) of bisexual men, 4.6% (n= 28) of lesbians, and 6.3% (n= 43) of bisexual women answered “yes.”

Compared to the general population who participated in the KNHANES VI (2013-2015), the prevalence of suicidal ideation over the past 12 months was 9.09-10.93 times higher among gay and bisexual men (gay men SPR, 9.09; 95% CI, 7.96 to 10.34, bisexual men SPR, 10.93; 95% CI, 7.61 to 15.20), 6.25-8.08 times higher among lesbians and bisexual women (SPR for lesbians, 6.25; 95% CI, 5.44 to 7.14, SPR for bisexual women, 8.08; 95% CI, 7.22 to 9.01) (Table 5). The suicide attempts were about 26-38 times more frequent in gay and bisexual men (SPR for gay men, 25.59; 95% CI, 17.61 to 35.94, SPR for bisexual men, 37.65; 95% CI, 12.23 to 87.86), and 7-10 times more frequent in lesbians and bisexual women (SPR for lesbian, 7.11; 95% CI, 4.72 to 10.27, SPR for bisexual women, 10.09; 95% CI, 7.30 to 13.60) than in the general population (Table 5).

In order to compare the prevalence of smoking and hazardous drinking of the Korean LGB adults with that of the general population, SPRs were calculated using data from 24,083 economically active men and 14,349 economically active women who participated in the 3rd KWCS (2011).

A total of 39.8% (n= 361) of gay men, 27.9% (n= 34) of bisexual men, 39.8% (n= 246) of lesbians, and 31.9% (n= 215) of bisexual women reported to be current smokers. When compared to the general population, the prevalence of smoking was about 6-7 times higher among lesbians and bisexual women (SPR for lesbians, 7.06; 95% CI, 6.21 to 8.00, SPR for bisexual women, 5.64; 95% CI, 4.91 to 6.45) (Table 6). In contrast, the prevalence of smoking among gay and bisexual men are statistically significantly lower than general population (SPR for gay men, 0.73; 95% CI, 0.65 to 0.81, SPR for bisexual men, 0.51; 95% CI, 0.36 to 0.72).

The prevalence of hazardous drinking was found to be 21.9% (n = 180) for gay men, 20.2% (n = 22) for bisexual men, 17.9% (n= 99) for lesbians, and 12.6% (n= 76) for bisexual women. When compared to the general population, prevalence of hazardous drinking was 2.18 times and 1.45 times higher among lesbians and bisexual women, respectively (SPR for lesbians, 2.18; 95% CI, 1.77 to 2.66, SPR for bisexual women, 1.45; 95% CI, 1.15 to 1.82), and 1.17 times higher among gay men (SPR, 1.17; 95% CI, 1.00 to 1.35) (Table 6).

## DISCUSSION

The present study showed that the overall health of the LGB adults in Korea is poorer than that of the general population. Compared to the general population, there was a higher prevalence of depressive symptoms, suicidal ideation and attempts, and musculoskeletal pains among Korean LGB adults. For women, the prevalence of reporting poor self-rated health and smoking was higher in the LGB population, and the prevalence of hazardous drinking was higher in the LGB population, with the exception of bisexual men, than in the general population. These findings are consistent with previous studies on health disparities of the LGB population [1-11].

The results about suicidal ideation and attempts in the Korean LGB adults particularly deserve attention. Our findings revealed that the prevalence of suicidal ideation among LGB adults was about 6.25 (lesbians) to 10.93 (bisexual men) times higher, and the prevalence of suicide attempts over the past 12 months was about 7.11 (lesbians) to 37.65 (bisexual men) times higher than in the general population. Korea has the highest suicide rate among Organization for Economic Cooperation and Development countries (29.1 suicides per 100,000 population) as of 2013 [22] and suicide is the main leading cause of death in Korean adults in their 20s-50s [23]. Considering these facts, our finding about suicidality of Korean LGB adults is very alarming.

To explain the health disparities of Korean LGB adults observed in this study, the minority stress processes in LGB population may be a useful framework [24]. Meyer [24] termed the stress experienced by sexual minorities in society due to their minority status as “minority stress” and theorized the process through which minority stress influences the mental health of sexual minorities. According to this model, all humans experience stressors in their daily lives but sexual minorities experience additional stressors leading to adverse health outcomes such as experiences of prejudice events, internalized homophobia, and expectations of rejection [24-29].

The minority stress model suggests that the hostile social environment for sexual minorities in Korea may be a key factor that exacerbates the health of sexual minorities. According to the Social Integration Survey in 2016, 55.8% of the respondents stated that they “do not accept” homosexuals [30]. Compared to the social acceptance toward other minority groups (North Korean defectors 12.1%, foreign immigrants or laborers 7.1%, people with disabilities 1.3%), the social acceptance for homosexuals is very low in Korea [30]. Furthermore, anti-lesbian, gay, bisexual, and transgender (LGBT) movement has been rapidly organized in Korea since the mid-2000s, and hate expressions against LGBT people have emerged as an important social problem [31-33]. In such social environment, various negative social experiences that sexual minorities face may worsen their health. Korean studies reported associations between social stigma and low life satisfaction in LGB adults [34] and between the experience of anti-gay violence and suicide risk in gay adolescents [35]. Further studies are needed to understand to what extent negative social experiences within the hostile environment of Korea explain for health disparities of LGB adults found in this study.

Another notable finding of this study is the differences in health status within the LGB population. Although the SPRs for mental health and physical symptoms against the general population were high in all the sexual minority groups, there were differences in self-rated health and health-related behaviors within the groups. The prevalence of poor self-rated health was higher in lesbians and bisexual women than in the general population, but no statistical difference was found among men. While lesbians and bisexual women showed a significantly higher prevalence of smoking than did the general population, gay and bisexual men showed a significantly lower prevalence of smoking than did the general population. Furthermore, SPRs for hazardous drinking was the highest in lesbians, followed by bisexual women and gay men, while there was no significant difference between bisexual men and the general population.

Previous studies also showed differences in health status among LGB population, but the findings were not consistent [1-5]. Further studies are needed to identify the factors and mechanisms behind health disparities within sexual minorities. More detailed examination on socio-demographic variables, such as gender, age, and socioeconomic status, is needed from an intersectional perspective before simply adjusting for them in the analysis. Additionally, more studies are needed to explore the health of lesbians and bisexual women. Both of them are relatively neglected populations in Korea LGBT health researches.

The present study has the following limitations. First, we cannot exclude the possibility that SPRs might be overestimated due to the difference in survey methods between our survey and the national health surveys used for the general population. Although the same questionnaires were used, we conducted an online survey while all the national-level health surveys were administered through face-to-face interview. Given the fact that sensitive questions, such as suicidal ideation and attempts, are more likely to be underreported in an in-person interview, it is difficult to completely exclude the possibility of overestimation of SPRs for suicidal behaviors. Second, there may be the possibility of potential under estimation of SPRs for musculoskeletal pains and healthrelated behaviors due to the difference in survey participants. Among our survey participants 33.5% were students, while participants of the KWCS used as a comparison group were limited to economically active population. Considering that the prevalence of musculoskeletal pains and health-related behaviors is generally higher in working populations, there is a possibility that the SPRs for those were underestimated. Third, the comparison group may have LGB individuals because those nationally representative surveys did not include sexual orientation measure in their questionnaires. Hence, it should be noted that the findings of this study were only a comparison of the health status of the Korean LGB adults with the general population, and not a comparison between LGB adults and their heterosexual counterparts.

Finally, the limitation of our study is that the representativeness of the sample cannot be ensured, as we used convenience sampling. The majority of participants were in their 20s-30s (90.9%) with high levels of education and income. It may be attributable to the fact that survey promotion and recruitment relied heavily on online and offline networks of sexual minority organizations and communities, as low-income and low-educated older LGB individuals may have lower access to such networks. In that case, there is a possibility that the SPRs in this study might be underestimated.

To address the limitations of this study and to gain a more systematic understanding of the health disparities of the Korean LGB population, nationally representative surveys should include sexual orientation in their questionnaires. A growing number of health surveys in North America and Europe include sexual orientation as a demographic variable, based on which many studies are conducted to investigate health disparities of sexual minorities [1-3,6,7,9,10,36,37]. The Korea Youth Risk Behavior Web-based Survey is the only national-level health survey in Korea measuring sexual orientation by asking respondents to indicate the gender of the person with whom they had a sexual intercourse. These data enabled studies to compare the health status of LGB adolescents with heterosexual counterparts [13-15]. To facilitate LGBT health studies in Korea, sexual orientation needs to be included as a demographic variable in national-level health surveys, such as the KNHANES and Work Environment Survey.

The present study aimed to investigate the magnitude of health disparities faced by the Korean LGB adults using the largest Korean dataset on LGB individuals. Future studies should take a step further to identify social factors that might worsen or improve their health. Furthermore, policy interventions should be implemented to improve the health and quality of life of sexual minorities in Korea.

## Figures and Tables

**Table 1.** Socio-demographic characteristics of participants by gender and sexual orientation (N=2,335)

	Total	Men (19-60 yr)		Women (19-50 yr)	
		Gay	Bisexual	Lesbian	Bisexual
Age (yr)^1^					
19-24	1,074 (46.1)	266 (29.1)	55 (45.1)	299 (48.3)	454 (66.7)
25-29	598 (25.6)	250 (27.4)	25 (20.5)	184 (29.7)	139 (20.4)
30-39	450 (19.3)	237 (26.0)	26 (21.3)	110 (17.8)	77 (11.3)
40-60	213 (9.1)	160 (17.5)	16 (13.1)	26 (4.2)	11 (1.6)
Education					
High school or less	199 (8.5)	77 (8.4)	12 (9.8)	56 (9.1)	54 (7.9)
College	282 (12.1)	105 (11.5)	10 (8.2)	87 (14.1)	80 (11.8)
University	1,591 (68.1)	594 (65.1)	89 (73.0)	416 (67.2)	492 (72.3)
Graduate school or higher	263 (11.3)	137 (15.0)	11 (9.0)	60 (9.7)	55 (8.1)
Household income (10,000 Korean won)^2^					
<1,000	397 (17.1)	131 (14.4)	24 (19.7)	107 (17.4)	135 (20.0)
1,000-1,999	364 (15.7)	136 (15.0)	21 (17.2)	94 (15.3)	113 (16.8)
2,000-2,999	430 (18.5)	180 (19.8)	18 (14.8)	104 (16.9)	128 (19.0)
3,000-3,999	366 (15.8)	147 (16.2)	17 (13.9)	96 (15.6)	106 (15.7)
4,000-4,999	248 (10.7)	96 (10.6)	18 (14.8)	68 (11.1)	66 (9.8)
≥5,000	516 (22.2)	220 (24.2)	24 (19.7)	146 (23.7)	126 (18.7)
Employment status^2^					
Permanent employee	590 (25.4)	299 (32.9)	31 (25.6)	145 (23.6)	115 (17.0)
Precarious employee	566 (24.4)	223 (24.6)	22 (18.2)	135 (22.0)	186 (27.4)
Employer	106 (4.6)	64 (7.1)	6 (5.0)	26 (4.2)	10 (1.5)
Unpaid family worker	24 (1.0)	11 (1.2)	2 (1.7)	6 (1.0)	5 (0.7)
Unemployed	259 (11.2)	96 (10.6)	13 (10.7)	75 (12.2)	75 (11.1)
College or university student	777 (33.5)	215 (23.7)	47 (38.8)	228 (37.1)	287 (42.3)
Residential area^2^					
Seoul Metropolitan City	1,113 (47.8)	482 (52.9)	53 (43.4)	271 (43.8)	307 (45.4)
Other metropolitan cities^3^	453 (19.5)	165 (18.1)	23 (18.9)	122 (19.7)	143 (21.2)
Other cities and counties	762 (32.7)	264 (29.0)	46 (37.7)	226 (36.5)	226 (33.4)
Total	2,335 (100.0)	913 (39.1)	122 (5.2)	619 (26.5)	681 (29.2)

Values are presented as number (%).

1 Men:19-60 yr, women: 19-50 yr.

2 Non-response: household income (n=14), employment status (n=13), and residential area (n=7).

3 Including Sejong Metropolitan Autonomous City.

**Table 2.** SPR of poor self-rated health^1^ between LGB population (2016) and the general population (2013-2015)^2^ in Korea (N=2,327)

		LGBadults		General population^2^	Age-SPR (95% CI)
		Total	Prevalence, n (%)	Prevalence (%)	
Men	Gay	911	111 (12.2)	12.0	1.10 (0.90, 1.32)
	Bisexual	119	9 (7.6)		0.69 (0.32, 1.31)
Women	Lesbian	618	138 (22.3)	14.2	1.80 (1.51, 2.13)
	Bisexual	679	184 (27.1)		2.24 (1.93, 2.59)

LGB, lesbian, gay, and bisexual; SPR, standardized prevalence ratio; CI, confidence interval.

1 Responses of ‘poor’ and ‘very poor’ were categorized as negative selfrated health.

2 Korea National Health and Nutrition Examination Survey VI (men: 19-60 yr; women: 19-50 yr).

**Table 3.** SPR of musculoskeletal pain between LGB population (2016) and the general population (2014)^1^ in Korea

			LGB adults		General population^1^	Age-SPR (95% CI)
			Total	Prevalence, n (%)	Prevalence (%)	
Back pain (n=2,322)	Men	Gay	907	214 (23.6)	12.9	2.85 (2.48, 3.25)
		Bisexual	120	30 (25.0)		3.40 (2.29, 4.85)
	Women	Lesbian	617	237 (38.4)	14.7	3.88 (3.41, 4.41)
		Bisexual	678	327 (48.2)		5.39 (4.83, 6.01)
Pain in the upper limbs (n=2,325)	Men	Gay	911	475 (52.1)	32.0	2.27 (2.07, 2.49)
		Bisexual	119	71 (59.7)		2.88 (2.25, 3.63)
	Women	Lesbian	617	471 (76.3)	34.4	3.08 (2.81, 3.37)
		Bisexual	678	532 (78.5)		3.42 (3.14, 3.73)
Pain in the lower limbs (n=2,324)	Men	Gay	909	283 (31.1)	19.3	2.30 (2.04, 2.58)
		Bisexual	121	46 (38.0)		2.97 (2.18, 3.97)
	Women	Lesbian	618	305 (49.4)	22.3	3.26 (2.91, 3.65)
		Bisexual	676	372 (55.0)		3.91 (3.52, 4.33)

SPR, standardized prevalence ratio; CI, confidence interval; LGB, lesbian, gay, and bisexual.

1 4th Korean Working Conditions Survey (men: 19-60 yr; women: 19-50 yr).

**Table 4.** SPR of depressive symptoms^1^ between LGB population (2016) and the general population (2015)^2^ in Korea (N=2,299)

		LGB adults		General population^2^	Age-SPR (95% CI)
		Total	Prevalence, n (%)	Prevalence (%)	
Men	Gay	899	305 (33.9)	5.6	7.13 (6.36, 7.98)
	Bisexual	118	43 (36.4)		7.16 (5.18, 9.65)
Women	Lesbian	610	288 (47.2)	7.3	5.08 (4.51, 5.70)
	Bisexual	672	398 (59.2)		6.12 (5.54, 6.76)

SPR, standardized prevalence ratio; LGB, lesbian, gay, and bisexual; CI, confidence interval.

1 Depressive symptoms were measured using the Center for Epidemiologic Studies-Depression Scale-D 11.

2 10th Korea Welfare Panel Study (men: 19-60 yr; women: 19-50 yr).

**Table 5.** SPR of suicidal ideation and suicide attempts over the past 12 months between LGB population (2016) and the general population (2013-2015)^1^ in Korea

			LGB adults		General population^1^	Age-SPR (95% CI)
			Total	Prevalence, n (%)	Prevalence (%)	
Suicidal ideation (n=2,325)	Men	Gay	907	233 (25.7)	3.5	9.09 (7.96, 10.34)
		Bisexual	122	35 (28.7)		10.93 (7.61, 15.20)
	Women	Lesbian	618	214 (34.6)	5.0	6.25 (5.44, 7.14)
		Bisexual	678	323 (47.6)		8.08 (7.22, 9.01)
Suicide attempts (n=2,322)	Men	Gay	908	33 (3.6)	0.3	25.59 (17.61, 35.94)
		Bisexual	120	5 (4.2)		37.65 (12.23, 87.86)
	Women	Lesbian	614	28 (4.6)	0.7	7.11 (4.72, 10.27)
		Bisexual	680	43 (6.3)		10.09 (7.30, 13.60)

SPR, standardized prevalence ratio; LGB, lesbian, gay, and bisexual; CI, confidence interval.

1 Korea National Health and Nutrition Examination Survey VI (men: 19-60 yr; women: 19-50 yr).

**Table 6.** SPR of smoking and hazardous drinking^1^ between LGB population (2016) and the general population (2011)^2^ in Korea

			LGB adults		General population^2^	Age-SPR (95% CI)
			Total	Prevalence, n (%)	Prevalence (%)	
Smoking (n=2,321)	Men	Gay	907	361 (39.8)	55.3	0.73 (0.65, 0.81)
		Bisexual	122	34 (27.9)		0.51 (0.36, 0.72)
	Women	Lesbian	618	246 (39.8)	5.7	7.06 (6.21, 8.00)
		Bisexual	674	215 (31.9)		5.64 (4.91, 6.45)
Hazardous drinking^1^ (n=2,089)	Men	Gay	823	180 (21.9)	21.6	1.17 (1.00, 1.35)
		Bisexual	109	22 (20.2)		1.12 (0.70, 1.70)
	Women	Lesbian	553	99 (17.9)	7.3	2.18 (1.77, 2.66)
		Bisexual	604	76 (12.6)		1.45 (1.15, 1.82)

SPR, standardized prevalence ratio; LGB, lesbian, gay, and bisexual; CI, confidence interval.

1 Following the Korean national guideline of the hazardous drinking, people who drink more than 7 drinks (for men) and 5 drinks (for women) per occasion and more than two time per week were classified as hazardous drinkers.

2 3rd Korean Working Conditions Survey (men: 19-60 yr; women: 19-50 yr).

## Appendix 1. SPR of 6 physical symptoms between LGB population (2016) and the general population (2014)^1^ in South Korea

**Table:**

			LGB adults		General population^1^	Age-SPR (95% CI)
			Total	Prevalence, n (%)	Prevalence (%)	
Hearing problems (n=2,322)	Men	Gay	907	78 (8.6)	1.8	7.41 (5.86, 9.25)
		Bisexual	121	11 (9.1)		7.96 (3.97, 14.24)
	Women	Lesbian	617	59 (9.6)	1.1	8.44 (6.42, 10.89)
		Bisexual	677	63 (9.3)		8.28 (6.36, 10.60)
Skin problems (n=2,325)	Men	Gay	907	316 (34.8)	2.2	17.32 (15.46, 19.33)
		Bisexual	120	45 (37.5)		17.56 (12.81, 23.50)
	Women	Lesbian	618	261 (42.2)	2.7	12.75 (11.25, 14.40)
		Bisexual	680	333 (49.0)		15.08 (13.50, 16.79)
Headaches/eyestrain (n=2,327)	Men	Gay	909	627 (69.0)	20.1	4.56 (4.21, 4.93)
		Bisexual	120	87 (72.5)		5.32 (4.26, 6.56)
	Women	Lesbian	618	524 (84.8)	22.7	4.34 (3.98, 4.73)
		Bisexual	680	604 (88.8)		4.83 (4.46, 5.23)
Stomach ache (n=2,321)	Men	Gay	907	280 (30.9)	1.4	23.78 (21.08, 26.74)
		Bisexual	120	35 (29.2)		22.78 (15.87, 31.68)
	Women	Lesbian	617	307 (49.8)	2.0	23.27 (20.74, 26.03)
		Bisexual	677	408 (60.3)		28.20 (25.53, 31.07)
Respiratory difficulties (n=2,324)	Men	Gay	908	77 (8.5)	0.7	17.09 (13.49, 21.36)
		Bisexual	120	9 (7.5)		15.16 (6.93, 28.77)
	Women	Lesbian	618	107 (17.3)	0.4	59.23 (48.54, 71.57)
		Bisexual	678	131 (19.3)		65.32 (54.62, 77.52)
Overall fatigue (n=2,326)	Men	Gay	910	549 (60.3)	24.5	3.28 (3.01, 3.56)
		Bisexual	121	74 (61.2)		3.64 (2.86, 4.57)
	Women	Lesbian	618	461 (74.6)	23.6	4.24 (3.87, 4.65)
		Bisexual	677	528 (78.0)		4.87 (4.47, 5.31)

SPR, standardized prevalence ratio; LGB, lesbian, gay, and bisexual; CI, confidence interval.

1 4th Korean Working Conditions Survey (men: 19-60 yr; women: 19-50 yr).
