# Supplementary material for: Health disparities between lesbian, gay, and bisexual adults and the general population in South Korea: Rainbow Connection Project I
Source: Epidemiol Health. 2017 Oct 19;39:e2017046. doi: 10.4178/epih.e2017046 (PMC5790982; doi:10.4178/epih.e2017046)
Supplement: Supplementary file 1 [file epih-39-e2017046-supplementary.pdf]

Open Access

Volume: 39, Article ID: e2017046
https://doi.org/10.4178/epih.e2017046

epiH Epidemiology and Health

ORIGINAL ARTICLE

한국 동성애자·양성애자의 건강불평등: 레인보우 커넥션 프로젝트 I

이호림[1], 이혜민[1], 박주영[1], 최보경[1], 김승섭[1,2]*

[1]고려대학교 일반대학원 보건과학과; [2]하바드대학교 보건대학원 사회행동학과

Health disparities between lesbian, gay, and bisexual adults and the general population in South Korea: Rainbow Connection Project I

Horim Yi[1], Hyemin Lee[1], Jooyoung Park[1], Bokyoung Choi[1], Seung-Sup Kim[1,2]*

[1]Graduate School of Korea University, Department of Public Health Sciences, Seoul, Korea; [2]Department of Social and Behavioral Sciences, Harvard T.H. Chan School of Public Health, Boston, MA, USA

Objectives: This study aims to investigate health disparities between lesbian, gay, and bisexual (LGB) adults and the general population in South Korea, where there is low public acceptance of sexual minorities and a lack of research on the health of sexual minorities.

Methods: The research team conducted a nationwide survey of 2,335 Korean LGB adults in 2016. Using the dataset, we estimated the age-standardized prevalence ratios (SPRs) for poor self-rated health, musculoskeletal pain, depressive symptoms, suicidal behaviors, smoking, and hazardous drinking. We then compared the SPRs of the LGB adults and the general population which participated in three different nationally representative surveys in Korea. SPRs were estimated for each of the four groups (i.e., gay men, bisexual men, lesbians, and bisexual women).

Results: Korean LGB adults exhibited a statistically significantly higher prevalence of depressive symptoms, suicidal ideation and attempts, and musculoskeletal pain than the general population. Lesbian and bisexual women had a higher risk of poor self-rated health and smoking than the general women population, whereas gay and bisexual men showed no differences with the general men population. Higher prevalence of hazardous drinking was observed among lesbians, gay men, and bisexual women compared to the general population, but was not observed in bisexual men.

Conclusions: The findings suggest that LGB adults have poorer health conditions compared to the general population in South Korea. These results suggest that interventions are needed to address the health disparities of Korean LGB adults.

KEY WORDS: Sexual minorities, Health status disparities, Minority health, Republic of Korea

Correspondence: Seung-Sup Kim
Department of Public Health Sciences, Graduate School of Korea University, 145 Anam-ro, Seongbuk-gu, Seoul 02841, Korea
E-mail: ssk3@korea.ac.kr

Received: Jul 5, 2017 / Accepted: Oct 19, 2017 / Published: Oct 19, 2017

This article is available from: http://e-epih.org/

This is an open-access article distributed under the terms of the Creative Commons Attribution License (http://creativecommons.org/licenses/by/4.0/), which permits unrestricted use, distribution, and reproduction in any medium, provided the original work is properly cited.

ⓒ 2017, Korean Society of Epidemiology

서 론[1]

성적지향(Sexual orientation)은 남성과 여성, 또는 양성 모두에 대한 정서적, 낭만적, 성적 끌림의 지속적인 패턴을 의미한다 [1]. 성소수자(Sexual Minority)로 일컬어지는 동성애자와 양성애자(Lesbian, Gay, Bisexual, LGB)는 성적지향의 차원에서 이성에게 성적인 끌림을 느끼는 이성애자와 달리 동성 또는 양성에게

[1]본 원고는 “Health Disparities Between Lesbian, Gay, and Bisexual Adults and the General Population in South Korea: Rainbow Connection Project I”의 한국어본으로 영문본과 달리 성소수자 인구집단에 대한 설명 등의 일부 내용이 추가되었고, 그에 따라 영문본과 인용문헌의 개수와 순서에도 차이가 있음을 알려드립니다.

성적인 끌림을 느끼는 이들을 말한다. 그동안 동성애자 및 양성애자 인구 규모는 킨제이(Alfred Kinsey)의 연구를 바탕으로 전체 인구 중 약 5%에서 10% 정도를 차지할 것으로 알려져 있었다[2]. 최근 미국에서 진행된 국가 대표성 있는 4개의 연구를 종합하여 성소수자 인구 규모를 추산한 바에 따르면, 동성애자와 양성애자, 트랜스젠더를 포함한 성소수자 인구는 사회 전체 인구의 약 2.2-5.6%를 차지할 것으로 추정된다[3].

세계적으로 동성애자·양성애자가 경험하는 건강불평등에 대한 다양한 연구가 진행되어 왔다. 이러한 선행연구들은 동성애자·양성애자가 일반인구에 비해 전반적으로 건강이 취약하다고 보고하고 있다[4-14]. 정신건강의 측면에서 동성애자와 양성애자는 전반적인 정신건강의 수준이 낮으며[6-8,13,14], 자살생각을 더 많이 하는 것[9]으로 나타났다. 신체적 건강의 측면에서도 동성애자·양성애자는 일반인구에 비해 불면증이나 피부염[4]을 포함한 여러 만성질환[6]의 유병률이 높은 것으로 나타났다. 또한 과음[11,12]이나 흡연[5,11,12] 등 건강위험행동 측면에서도 동성애자·양성애자가 일반인구에 비해 높은 위험을 가진다고 알려져 있다.

그러나 한국 동성애자와 양성애자의 건강에 대한 연구는 매우 부족한 상황이다. Lee 등(2014)이 진행한 체계적 문헌고찰 논문에 따르면, 2013년까지 학술지에 출판된 한국 성소수자 건강 연구는 123편에 불과하다[15]. 그 중 75%에 해당하는 101편은 임상적 논문으로, 대부분 의료기관에서 트랜스젠더 및 간성(Intersex)을 대상으로 한 성전환 수술에 대한 사례보고 연구였다. 반면, 123편 중 동성애자·양성애자 건강 연구는 30편에 불과했으며, 이들의 건강불평등이나 건강에 영향을 줄 수 있는 사회적 요인을 다룬 연구는 25편으로 매우 제한적이었다. 사회적 요인을 다룬 연구의 대부분은 성소수자 정체성 관련 스트레스 요인과 정신건강의 연관성을 파악하고자 했다.

기존의 한국 동성애자·양성애자 건강불평등 연구는 그 숫자가 매우 적으며, 출판된 연구들 역시 다음과 같은 한계를 가지고 있다. 첫째, 대부분의 동성애자·양성애자 건강불평등 연구는 성적지향 관련 변수를 가지고 있는 국가 대표성을 지닌 설문조사인 ‘청소년온라인건강행태조사’를 활용한 청소년 대상 연구였다[16-18]. 반면, 성인을 참여대상으로 하는 국가 대표성 있는 설문조사 중에는 성적지향 관련 변수를 포함하는 조사가 없기 때문에 성인 동성애자·양성애자 건강불평등 연구는 상대적으로 거의 진행되지 못했다. 둘째, 양성애자 및 여성에 대한 연구가 전무하다. 기존에 진행된 한국 성인 동성애자·양성애자의 건강불평등 연구는 남성 동성애자 또는 남성과 성관계를 가지는 남성(Men who have sex with men, MSM)만을 연구대상으로 삼았고[19-22], MSM 대상 연구는 동성애자와 양성애자를 구분하지 않았다[19]. 따라서, 현재까지 한국 여성 동성애자 및 남녀 양성애자의 건강상태를 일반인구 집단과 비교한 연구는 존재하지 않는다. 남성 동성애자·양성애자 성인과 일반인구의 건강수준을 비교한 연구들은 성인 동성애자·양성애자와 비교 대상 인구집단을 별도로 표집하여 이들의 건강상태를 비교하였으며[19-22], 동성애자·양성애자 연구참여자 수는 최대 129명에 불과했다. 마지막으로 기존 동성애자·양성애자 건강불평등 연구에서 다룬 건강 변수는 정신건강과 건강행동 일부에 제한되어 있다. 기존 연구들은 한국 일반인구에 비해 동성애자·양성애자가 음주, 흡연 등 건강위험행동을 할 가능성이 높고[17], 우울수준[17,20,23]과 자살위험[18,20]이 높다고 보고하였으나, 이들의 신체적 건강을 다룬 연구는 전무했다.

본 연구는 만 19세 이상의 한국 성인 동성애자와 양성애자를 대상으로 이들의 건강상태에 대한 설문조사를 시행하고, 수집한 자료를 분석하여 동성애자·양성애자의 건강불평등 실태를 파악하고자 하였다. 본 연구의 목적은 자가평가건강, 정신건강(우울, 자살생각 및 자살시도), 신체적 건강(근골격계 통증) 및 건강관련 행동(흡연율, 위험음주율)을 포함하여 다양한 측면에서 한국 성인 동성애자·양성애자와 일반인구집단의 건강수준을 비교하는 것이다.

연구방법

분석대상과 자료

본 연구는 한국 동성애자·양성애자의 사회적 경험과 건강을 이해하기 위해 ‘레인보우 커넥션 프로젝트 I - 한국 성인 레즈비언 게이 바이섹슈얼 건강 연구’라는 제목으로 한국에 거주하는 만 19세 이상의 성인 동성애자와 양성애자가 참여하는 설문조사를 시행하였다. 성소수자는 주류와 다른 성적지향이나 성별정체성을 지닌 이들을 아우르는 용어로 동성애자 및 양성애자뿐만 아니라 트랜스젠더 등을 포함하지만, 본 연구에서는 연구참여자를 성소수자 중 동성애자와 양성애자로 한정하였다. 성별정체성 차원에서 소수자 정체성을 지닌 트랜스젠더의 경우 동성애자나 양성애자와 사회적 경험 및 건강 측면에서 다른 특성을 보일 수 있기 때문이다. 실제 성소수자와 관련한 국내외 선행 연구에서도 동성애자 및 양성애자와 트랜스젠더에 대한 연구를 별도로 진행하는 경우가 많다[15, 24].

설문조사는 2016년 11월 11일부터 12월 5일까지 약 4주간 온라인 설문조사를 통해 진행되었다. 설문조사 홍보와 연구 참여자 모집은 한국의 성소수자들이 많이 이용하는 웹사이트 2곳과 성소수자 인권단체의 페이스북(Facebook), 트위터(Twitter) 등 소셜 네트워크 서비스 계정을 통해 진행되었다. 본 연구는 고려대학교 기관연구윤리심의위원회의 승인을 받았으며(승인번호: 1040548-KU-IRB-16-191-A-1), 모든 연구참여자에게 연구참여와 학술 활용에 대해 IRB의 승인을 받은 방식으로 동의를 받은 후 설문조사를 진행하였다. 모든 연구참여자에게는 연구참여에 대한 보상으

로 5천원 상당의 리워드를 제공하였다.

설문조사에 참여한 총 인원은 2,430명이었으며, 연구 참여와 학술 활용에 동의하지 않은 17명과 인구사회학적 정보에 응답하지 않은 72명을 제외하였다. 또한 남성의 경우 61세 이상, 여성의 경우 51세 이상 연령대의 연구참여자가 너무 적어 각각 5명과 1명을 제외하였다. 결과적으로 총 2,335명의 자료를 본 연구에 활용하였다.

측정도구

설문지는 인구사회학적 정보, 성적지향 관련 정보, 사회적 경험, 건강상태 등 4가지 범주의 총 173개 문항으로 구성되었다.

자가평가건강수준은 "매우 좋다", "좋은 편이다", "보통이다", "나쁜 편이다", "매우 나쁘다"의 5점 척도로 측정하였으며, "나쁜 편이다"와 "매우 나쁘다"라는 응답을 자가평가건강 수준이 나쁜 집단으로 분류하였다. 신체적 건강 수준을 확인하기 위한 변수로는 총 9가지 신체적 증상을 조사하였고, 본 연구에서는 근골격계 통증의 결과만을 포함하였다. 근골격계 통증은 총 3문항으로 지난 12개월 동안 a) 요통, b) 어깨, 목, 팔 등 상지 근육통, c) 엉덩이, 다리, 무릎, 발 등 하지 근육통이 있었는지 여부에 대해 "있다", "없다"로 응답하도록 했다. 근골격계 증상을 제외한 6가지 신체적 증상에 대한 결과는 부록 1에서 확인할 수 있다

우울 증상의 경우 설문조사에서는 The Center for Epidemiologic Studies-Depression Scale-D (CES-D) 20 척도로 측정하였으나, 비교 대상 자료인 한국복지패널에서 CES-D 11을 활용하였기 때문에 본 연구의 목적인 건강불평등 분석을 위해 CES-D 20의 일부인 CES-D 11을 이용하였다. CES-D 11은 0-3점까지의 4점 척도로 각 문항별 점수를 합한 총점(33점)에 20/11을 곱하여 60점으로 환산한 후 16점 이상인 경우를 우울증상을 가지는 것으로 분류하였다. 또한 자살 생각과 시도의 경우 각각 한 문항으로 지난 12개월간 자살생각("귀하는 지난 12개월 동안 자살하는 것에 대해 진지하게 생각한 적이 한 번이라도 있습니까?")과 자살시도("귀하는 지난 12개월 동안 자살을 시도한 적이 있습니까?") 여부를 측정하였다.

흡연의 경우, 평생 흡연량과 현재 흡연여부를 측정하고 이를 활용하여, '평생 담배 5갑(100개비) 이상 피웠고 현재 담배를 피우는 이들'을 흡연자로 분류하였다. 위험음주는 음주빈도와 음주량을 측정하고 이를 바탕으로 위험음주에 대한 한국의 국가 가이드라인[25]에 따라 '현재 음주를 하는 이들 중 주 2회 이상, 1회 평균 음주량이 남성 7잔, 여성 5잔 이상' 시 위험음주로 분류하였다.

분석방법

본 연구는 한국 동성애자·양성애자의 건강상태와 건강행동 분포를 확인하고, 이를 일반인구집단과 비교하였다. 비교 대상 자료로는 본 연구의 설문조사와 건강 관련 변수의 측정방식과 준거기간이 동일한 국가 대표성 있는 건강조사 중 본 연구의 설문조사 시기와 가장 근접한 시기에 조사된 자료를 활용하였다. 구체적으로, 자가평가건강과 지난 12개월 간 자살생각 및 시도는 국민건강영양조사(제6기), 근골격계 통증은 근로환경조사(4차), 우울증상은 한국복지패널(제10차), 흡연과 위험음주는 근로환경조사(3차)의 자료를 활용하였다.

동성애자·양성애자와 일반인구집단의 건강 수준을 비교하기 위해 연령에 대한 간접표준화(Indirect standardization)를 실시하였다. 이에 따라 성인 동성애자·양성애자 집단의 관측된 케이스 수(Observed number of cases)와 일반 인구집단의 연령대별 유병률을 성인 동성애자·양성애자 집단의 연령대별 참여자 수에 적용하여 기대 된 케이스 수(Expected number of cases)의 비(ratio)인 표준화유병비(Standardized Prevalence Ratio, SPR)를 산출하였다. 이는 일반인구와 동성애자·양성애자 인구의 연령분포상 차이로 인한 편의를 방지하기 위함이다. 일반인구집단의 연령별 유병률 산출 과정에서는 Stata의 svyset과 svy 명령어를 활용하여 각 조사별 개인 횡단면 가중치를 부여해 모집단을 대표하는 유병률을 구하였다. 또한 성별과 성적지향에 따른 특성을 고려하기 위해, 성별(남/여)과 성적지향(동성애/양성애)에 따른 네 집단(i.e. 동성애자 남성, 양성애자 남성, 동성애자 여성, 양성애자 여성)으로 나누었다. 이에 따라 동성애자 남성과 양성애자 남성은 남성 일반인구와, 동성애자 여성과 양성애자 여성은 여성 일반인구와 비교한 표준화유병비를 각각 산출하였다. 모든 통계적 분석에는 STATA/SE version 13.0을 활용하였다.

결 과

인구사회학적 특성

본 연구에 참여한 연구참여자들의 인구학적 특성은 표 1과 같다. 연구참여자의 성별과 성적지향은 동성애자 남성이 39.1% (N=913), 양성애자 남성이 5.2% (N=122), 동성애자 여성이 26.5% (N=619), 양성애자 여성이 29.2% (N=681)로 나머지 세 집단에 비해 양성애자 남성의 참여가 적은 편이었다. 연령은 19세 이상에서 39세 이하가 90.9% (N=2,122), 40세 이상이 9.1% (N=213)였다. 교육수준의 경우 4년제 대학교 재학, 중퇴, 졸업이 68.1% (N=1,591)로 가장 많은 비율을 차지했으며, 전문대 재학·중퇴·졸업(12.1%, N=282), 대학원 이상(11.3%, N=263), 고졸 이하(8.5%, N=199) 순이었다. 가구 소득은 5,000만원 이상이 22.2% (N=516)로 가장 많은 비율을 차지했다. 고용형태의 경우 현재 학생으로 현재 경제활동인구가 아닌 이들이 33.5% (N=777), 정규직 임금근로자가 25.4% (N=590), 기간제·시간제·계약직·특수형태근로 등을 포함한 비정규직 임금근로자가 24.4% (N=566), 실업 또는 무직이 11.2% (N=259), 자영업자 또는 고용주가 4.6% (N=106), 무급가족종사자가 1.0%

표 1. 한국 성인 동성애자·양성애자의 성별과 성적 지향에 따른 인구사회학적 특성 (N=2,335)

	전체 N (%)	남성(19-60세)		여성(19-50세)	
		동성애자 남성 N (%)	양성애자 남성 N (%)	동성애자 여성 N (%)	양성애자 여성 N (%)
연령[1]					
19-24	1,074 (46.1)	266 (29.1)	55 (45.1)	299 (48.3)	454 (66.7)
25-29	598 (25.6)	250 (27.4)	25 (20.5)	184 (29.7)	139 (20.4)
30-39	450 (19.3)	237 (26.0)	26 (21.3)	110 (17.8)	77 (11.3)
40-60[1]	213 (9.1)	160 (17.5)	16 (13.1)	26 (4.2)	11 (1.6)
교육수준					
고등학교 졸업 이하	199 (8.5)	77 (8.4)	12 (9.8)	56 (9.1)	54 (7.9)
전문대(2년제) 재학, 중퇴, 졸업	282 (12.1)	105 (11.5)	10 (8.2)	87 (14.1)	80 (11.8)
대학교(4년제) 재학, 중퇴, 졸업	1,591 (68.1)	594 (65.1)	89 (73.0)	416 (67.2)	492 (72.3)
대학원 이상	263 (11.3)	137 (15.0)	11 (9.0)	60 (9.7)	55 (8.1)
연평균 가구소득[2]					
1천만원 미만	397 (17.1)	131 (14.4)	24 (19.7)	107 (17.4)	135 (20.0)
1-2천만원 미만	364 (15.7)	136 (15.0)	21 (17.2)	94 (15.3)	113 (16.8)
2-3천만원 미만	430 (18.5)	180 (19.8)	18 (14.8)	104 (16.9)	128 (19.0)
3-4천만원 미만	366 (15.8)	147 (16.2)	17 (13.9)	96 (15.6)	106 (15.7)
4-5천만원 미만	248 (10.7)	96 (10.6)	18 (14.8)	68 (11.1)	66 (9.8)
5천만원 이상	516 (22.2)	220 (24.2)	24 (19.7)	146 (23.7)	126 (18.7)
고용형태[2]					
정규직 임금근로자	590 (25.4)	299 (32.9)	31 (25.6)	145 (23.6)	115 (17.0)
비정규직 임금근로자	566 (24.4)	223 (24.6)	22 (18.2)	135 (22.0)	186 (27.4)
자영업자 또는 고용주	106 (4.6)	64 (7.1)	6 (5.0)	26 (4.2)	10 (1.5)
무급가족종사자	24 (1.0)	11 (1.2)	2 (1.7)	6 (1.0)	5 (0.7)
실업상태 또는 무직	259 (11.2)	96 (10.6)	13 (10.7)	75 (12.2)	75 (11.1)
학생	777 (33.5)	215 (23.7)	47 (38.8)	228 (37.1)	287 (42.3)
거주 지역[2]					
서울특별시	1,113 (47.8)	482 (52.9)	53 (43.4)	271 (43.8)	307 (45.4)
광역시[3]	453 (19.5)	165 (18.1)	23 (18.9)	122 (19.7)	143 (21.2)
그 외 지역	762 (32.7)	264 (29.0)	46 (37.7)	226 (36.5)	226 (33.4)
전체	2,335 (100.0)	913 (39.1)	122 (5.2)	619 (26.5)	681 (29.2)

[1]남성 19-60, 여성 19-50; [2]무응답-연평균 가구소득 (N=14), 고용형태 (N=13), 거주지역 (N=7); [3]세종특별자치시 포함.

표 2. 한국 성인 동성애자·양성애자(2016)와 일반 인구(2013-2015)[1]의 자가평가 건강수준[2] 표준화유병비 (N=2,327)

		성인 동성애자·양성애자		일반 인구[1]	연령 표준화유병비	
		전체	유병률 N (%)	유병률 (%)	SPR	95% CI
남성	동성애자	911	111 (12.2)	12.0	1.10	0.90, 1.32
	양성애자	119	9 (7.6)		0.69	0.32, 1.31
여성	동성애자	618	138 (22.3)	14.2	1.80	1.51, 2.13
	양성애자	679	184 (27.1)		2.24	1.93, 2.59

SPR, standardized prevalence ratio, 표준화유병비; CI, confidence interval, 신뢰구간.
[1]국민건강영양조사 6기(남성: 19-60세; 여성 19-50세); [2]'나쁜 편이다'와 '매우 나쁘다'를 부정적 자가평가건강으로 분류하였음.

(N=24) 순이었다. 거주지역은 서울이 47.8% (N=1,113)로 가장 많은 비율을 차지했고, 광역시 19.5% (N=453), 그 외 시·군 지역이 32.7% (N=762)로 나타났다.

한국 성인 동성애자·양성애자와 일반 인구의 건강상태 비교

표 2에 따르면, 자가평가건강수준에 대해 동성애자 남성의 12.2% (N=111), 양성애자 남성의 7.6% (N=9), 동성애자 여성의 22.3% (N=138), 양성애자 여성의 27.1% (N=184)가 자신의 건강상태를 "나쁜 편이다" 또는 "매우 나쁘다"로 보고하였다.

한국 성인 동성애자·양성애자의 부정적 자가평가건강의 빈도를 일반인구와 비교하기 위해 국민건강영양조사 6기(2013-2015)에 참여한 일반 인구 중 19-60세에 속하는 남성 4,546명과 19-50세에 속하는 여성 4,432명의 자료를 이용하여 표준화유병비를 산출하였다. 그 결과, 성별 및 연령 구조가 동일한 일반인구 집단과 비교하였을 때, 동성애자 여성 (SPR [표준화유병비]: 1.80, 95% CI [신뢰구간]: 1.51-2.13)과 양성애자 여성(SPR: 2.24, 95% CI: 1.93-2.59)의 경우 부정적인 자가평가건강 빈도가 각각 여성 일반인구에 비해 1.80배와 2.24배 높게 나타났다. 그러나 동성애자 남성과 양성애자 남성의 경우 남성 일반인구 집단과 통계적으로 유의한

표 3. 한국 성인 동성애자·양성애자(2016)와 일반 인구(2014)[1]의 근골격계 통증 표준화유병비

			성인 동성애자·양성애자		일반 인구[1]	연령 표준화유병비	
			전체	유병률 N (%)	유병률(%)	SPR	95% CI
요통(N=2,322)	남성	동성애자	907	214 (23.6)	12.9	2.85	2.48, 3.25
		양성애자	120	30 (25.0)		3.40	2.29, 4.85
	여성	동성애자	617	237 (38.4)	14.7	3.88	3.41, 4.41
		양성애자	678	327 (48.2)		5.39	4.83, 6.01
상지통(N=2,325)	남성	동성애자	911	475 (52.1)	32.0	2.27	2.07, 2.49
		양성애자	119	71 (59.7)		2.88	2.25, 3.63
	여성	동성애자	617	471 (76.3)	34.4	3.08	2.81, 3.37
		양성애자	678	532 (78.5)		3.42	3.14, 3.73
하지통(N=2,324)	남성	동성애자	909	283 (31.1)	19.3	2.30	2.04, 2.58
		양성애자	121	46 (38.0)		2.97	2.18, 3.97
	여성	동성애자	618	305 (49.4)	22.3	3.26	2.91, 3.65
		양성애자	676	372 (55.0)		3.91	3.52, 4.33

SPR, standardized prevalence ratio, 표준화유병비; CI, confidence interval, 신뢰구간.
[1]제4차 근로환경조사(남성: 19-60세, 여성 19-50세).

차이가 나타나지 않았다(표 2).

한국 성인 동성애자·양성애자가 지난 12개월 동안 경험한 근골격계 통증 빈도를 일반인구와 비교하기 위해 2014년 제4차 근로환경조사에 참여한 경제활동인구 중 19-60세에 속하는 남성 20,075명과 19-50세에 속하는 여성 14,066명의 자료를 이용하여 간접표준화를 실시하였다. 그 결과를 표준화된 일반인구 집단과 비교하였을 때, 동성애자 남성, 양성애자 남성, 동성애자 여성, 양성애자 여성 모두 요통, 상지통, 하지통 빈도가 통계적으로 유의하게 높은 것으로 나타났다. 예를 들어 요통의 경우, 남성 경제활동인구에 비해 동성애자 남성(SPR 2.85, 95% CI: 2.48-3.25), 양성애자 남성(SPR 3.40, 95% CI: 2.29-4.85)이 2.85배에서 3.40배 높은 요통 유병률을 가지는 것으로 나타났다. 동성애자 여성(SPR 3.88, 95% CI: 3.41-4.41)과 양성애자 여성(SPR 5.39, 95% CI: 4.83-6.01)은 여성 경제활동인구에 비해 3.88배에서 5.39배까지 요통 유병률이 높았다(표 3).

표 4는 동성애자 남성 33.9% (N=305)와 양성애자 남성 36.4% (N=43), 동성애자 여성 47.2% (N=288), 양성애자 여성 59.2% (N=398)가 우울증상을 경험하고 있었음을 보여준다.

이를 일반인구 집단과 비교하기 위해 제10차 한국복지패널조사(2015)에 참여한 일반인구 중 19-60세에 속하는 남성 3,308명과 19-50세에 속하는 여성 2,705명의 자료를 활용하여 표준화유병비를 산출하였다. 분석 결과, 동성애자·양성애자 집단의 우울증상 유병률은 일반인구 집단보다 동성애자 남성(SPR 7.13, 95% CI: 6.36-7.98)과 양성애자 남성(SPR 7.16, 95% CI: 5.18-9.65)에서 7.13배에서 7.16배 높은 것으로 나타났으며, 동성애자 여성(SPR 5.08, 95% CI: 4.51-5.70)과 양성애자 여성(SPR 6.12, 95% CI: 5.54-6.76)에서 5.08배에서 6.12배까지 높은 것으로 확인되었다(표 4).

표 4. 한국 성인 동성애자·양성애자(2016)와 일반 인구(2015)[1]의 우울증상[2] 표준화유병비(N=2,299)

		성인 동성애자·양성애자		일반 인구[1]	연령 표준화유병비	
		전체	유병률 N (%)	유병률 (%)	SPR	95% CI
남성	동성애자	899	305 (33.9)	5.6	7.13	6.36, 7.98
	양성애자	118	43 (36.4)		7.16	5.18, 9.65
여성	동성애자	610	288 (47.2)	7.3	5.08	4.51, 5.70
	양성애자	672	398 (59.2)		6.12	5.54, 6.76

SPR, standardized prevalence ratio, 표준화유병비; CI, confidence interval, 신뢰구간.
[1]제10차 한국복지패널조사(남성: 19-60세, 여성: 19-50세); [2]우울증상은CES-D 11로 측정함.

"지난 12개월 동안 자살하는 것에 대해 진지하게 생각한 적이 한 번이라도 있"었다고 응답한 이는 동성애자 남성 25.7% (N=233), 양성애자 남성 28.7% (N=35), 동성애자 여성 34.6% (N=214), 양성애자 여성 47.6% (N=323)였다. 또한 "지난 12개월 동안 자살을 시도한 적이 있"었다고 응답한 이는 동성애자 남성 3.6% (N=33), 양성애자 남성 4.2% (N=5), 동성애자 여성 4.6% (N=28), 양성애자 여성 6.3% (N=43)였다.

이를 국민건강영양조사 6기(2013-2015)에 참여한 일반인구 집단과 비교한 결과, 동성애자 남성과 양성애자 남성의 지난 12개월간 자살생각의 빈도는 일반 남성에 비해 9.09배에서 10.93배(동성애자 남성 SPR 9.09, 95% CI: 7.96-10.34, 양성애자 남성 SPR 10.93, 95% CI: 7.61-15.20)까지 높은 것으로 나타났으며, 동성애자 여성과 양성애자 여성의 지난 12개월간 자살생각의 빈도는 일반 여성에 비해 6.25배에서 8.08배(동성애자 여성 SPR 6.25, 95% CI: 5.44-

표 5. 한국 성인 동성애자·양성애자(2016)와 일반 인구(2013-2015)[1]의 지난 12개월 자살생각 및 자살시도의 표준화유병비

			성인 동성애자·양성애자		일반 인구[1]	연령 표준화유병비	
			전체	유병률 N (%)	유병률(%)	SPR	95% CI
자살생각(N=2,325)	남성	동성애자	907	233 (25.7)	3.5	9.09	7.96, 10.34
		양성애자	122	35 (28.7)		10.93	7.61, 15.20
	여성	동성애자	618	214 (34.6)	5.0	6.25	5.44, 7.14
		양성애자	678	323 (47.6)		8.08	7.22, 9.01
자살시도(N=2,322)	남성	동성애자	908	33 (3.6)	0.3	25.59	17.61, 35.94
		양성애자	120	5 (4.2)		37.65	12.23, 87.86
	여성	동성애자	614	28 (4.6)	0.7	7.11	4.72, 10.27
		양성애자	680	43 (6.3)		10.09	7.30, 13.60

SPR, standardized prevalence ratio, 표준화유병비; CI, confidence interval, 신뢰구간.
[1]국민건강영양조사6기(남성: 19-60세, 여성: 19-50세).

표 6. 한국 성인 동성애자·양성애자(2016)와 일반 인구(2011)[1]의 흡연과 위험음주[2]의 표준화유병비

			성인 동성애자·양성애자		일반 인구[1]	연령 표준화유병비	
			전체	유병률 N (%)	유병률(%)	SPR	95% CI
흡연(N=2,321)	남성	동성애자	907	361 (39.8)	55.3	0.73	0.65, 0.81
		양성애자	122	34 (27.9)		0.51	0.36, 0.72
	여성	동성애자	618	246 (39.8)	5.7	7.06	6.21, 8.00
		양성애자	674	215 (31.9)		5.64	4.91, 6.45
위험음주[2] (N=2,089)	남성	동성애자	823	180 (21.9)	21.6	1.17	1.00, 1.35
		양성애자	109	22 (20.2)		1.12	0.70, 1.70
	여성	동성애자	553	99 (17.9)	7.3	2.18	1.77, 2.66
		양성애자	604	76 (12.6)		1.45	1.15, 1.82

SPR, standardized prevalence ratio, 표준화유병비; CI, confidence interval, 신뢰구간.
[1]제3차 근로환경조사(남성: 19-60세, 여성: 19-50세); [2]위험음주는 1회 평균 음주량이 남성은 7잔 이상, 여성은 5잔 이상을 주 2회 이상 음주하는 경우를 의미함.

7.14, 양성애자 여성 SPR 8.08, 95% CI: 7.22-9.01) 높은 것으로 확인되었다(표 5). 지난 12개월간 자살시도의 빈도는 일반인구 집단에 비해 동성애자 남성과 양성애자 남성 집단은 약 26-38배(동성애자 남성 SPR 25.59, 95% CI: 17.61-35.94, 양성애자 남성 SPR 37.65, 95% CI: 12.23-87.86), 동성애자 여성과 양성애자 여성 집단은 약 7-10배(동성애자 여성 SPR 7.11, 95% CI: 4.72-10.27, 양성애자 여성 SPR 10.09, 95% CI: 7.30-13.60) 높게 나타났다(표 5).

한국 성인 동성애자·양성애자의 흡연율과 위험음주율을 일반인구와 비교하기 위해 2011년 제3차 근로환경조사에 참여한 경제활동인구 중 19-60세에 속하는 남성 24,083명과 19-50세에 속하는 여성 14,349명의 자료를 활용하여 표준화유병비를 산출하였다.

흡연율의 경우, 동성애자 남성 39.8% (N=361), 양성애자 남성 27.9% (N=34), 동성애자 여성 39.8% (N=246), 양성애자 여성 31.9% (N=215)가 현재 흡연을 하고 있다고 보고하였다. 이를 제3차 근로환경조사에 참여한 일반인구 집단과 비교한 결과, 흡연율은 일반인구 집단에 비해 동성애자 및 양성애자 여성이 일반인구 여성 집단에 비해 약 6-7배(동성애자 여성 SPR 7.06, 95% CI: 6.21-8.00, 양성애자 여성 SPR 5.64, 95% CI: 4.91-6.45)정도 높게 나타났다(표 6). 반면, 동성애자·양성애자 남성의 흡연율은 일반인구 집단의 0.51-0.73배 수준으로 통계적으로 유의하게 낮은 것으로 나타났다(동성애자 남성 SPR 0.73, 95% CI: 0.65-0.81, 양성애자 남성 SPR 0.51, 95% CI: 0.36-0.72).

동성애자·양성애자의 위험음주율은 동성애자 남성 21.9% (N=180), 양성애자 남성 20.2% (N=22), 동성애자 여성 17.9% (N=99), 양성애자 여성 12.6% (N=76)로 나타났다. 이를 제3차 근로환경조사(2011) 자료를 활용해 일반인구 집단과 비교한 결과, 동성애자 및 양성애자 여성은 여성 일반인구에 비해 위험음주율이 각각 2.18배, 1.45배(동성애자 여성 SPR 2.18, 95% CI: 1.77-2.66, 양성애자 여성 SPR 1.45, 95% CI: 1.15-1.82) 높게 나타났으며, 동성애자 남성의 경우 남성 일반인구에 비해 1.17배(SPR 1.17, 95% CI:

1.00-1.35) 높은 것으로 나타났다(표 6).

고 찰

본 연구는 한국 성인 동성애자·양성애자의 전반적인 건강 수준이 일반인구에 비해 취약한 수준임을 보여준다. 한국 성인 남녀 동성애자·양성애자 모두 일반인구에 비해 높은 우울증상과 자살생각 및 시도, 근골격계 통증 모두에서 높은 유병률을 보였다. 여성의 경우 부정적 자가평가건강의 보고와 흡연율도 일반인구에 비해 높은 수준이었으며, 양성애자 남성을 제외한 모든 성소수자 집단의 위험음주율도 일반 인구에 비해 높은 것으로 나타났다. 이러한 연구 결과는 외국에서 진행된 기존의 동성애자·양성애자 건강불평등 연구의 결과와 유사하다[4-14].

특히 본 연구에서 한국 동성애자·양성애자의 자살생각과 자살시도에 대한 결과는 주목할 필요가 있다. 연구 결과, 동성애자·양성애자의 지난 12개월간 자살생각 유병률은 국민건강영양조사 6기(2013-2015)에 참여한 일반인구에 비해 약 6.25배(동성애자 여성)에서 10.93배(양성애자 남성)까지 높은 수준이었으며, 지난 12개월간 자살시도도 7.11배(동성애자 여성)에서 37.65배(양성애자 남성)까지 높은 수준이었다. 비교집단인 한국 일반인구의 자살률이 2013년 기준으로 OECD 국가 중 가장 높은 100,000명 당 29.1명이라는 점[26]과 한국 20세부터 39세까지 사망원인의 1위 그리고 40세부터 59세까지 사망원인의 2위가 자살이라는 점[27]을 고려할 때, 본 연구에서 드러난 한국 동성애자·양성애자의 자살위험성은 매우 우려되는 상황이다.

이와 같은 한국 동성애자·양성애자의 건강불평등을 설명하기 위해서는 성소수자에게 적대적인 사회환경으로 인해 성소수자가 경험하는 소수자 스트레스 과정 모형(Minority stress processes in lesbian, gay, and bisexual populations)[28]이 유용할 수 있다. 미국의 공중보건학자 Ilan Meyer는 성소수자가 사회환경에서 자신의 소수자 지위로 인해 경험하는 스트레스를 소수자 스트레스(Minority stress)로 명명하고, 이러한 소수자 스트레스가 성소수자의 정신건강에 영향을 미치는 과정을 이론화하였다[28]. 이 모형에 따르면, 모든 인간은 일상생활에서 스트레스를 경험하지만, 성소수자는 추가적으로 자신의 소수자 지위로 인해 차별이나 폭력 등 편견적 사건 경험과 배제에 대한 예상, 정체성에 대한 숨김, 내재화된 동성애혐오 등의 소수자 스트레스를 경험한다. 이 모델에 따른 선행연구들은 실제 성소수자 정체성으로 인해 겪을 수 있는 다양한 부정적인 사회적 경험이 동성애자·양성애자의 건강불평등과 연관성을 가지는 요인 중 하나임을 제시하고 있다[29-33]. 성적지향에 대한 부정적인 태도를 더 많이 경험하는 동성애자·양성애자들이 그렇지 않은 이들에 비해 정신건강 문제를 더 많이 경험하는 것으로 나타났으며[29], 성인 동성애자·양성애자의 정신건강이 나쁜 이유가 청소년기의 따돌림 경험과 같은 어린 시절 부정적인 사회적 경험 때문이라는 연구결과도 존재한다[31]. 나아가 신체적 건강 측면에서도 성소수자에 대한 편견에 기반한 부정적 경험을 한 성인 동성애자·양성애자가 그렇지 않은 이들에 비해 1년 후 신체적 건강 문제를 더 많이 경험한다는 종단연구 결과가 보고된 바 있다[30].

소수자 스트레스 모형은 성소수자에게 적대적인 한국의 사회환경이 성소수자의 건강을 악화시키는 주요한 원인일 수 있음을 제시하고 있다. 2016년 사회통합실태조사에 따르면, 응답자의 55.8%가 동성애자를 “받아들일 수 없다”고 응답했다[34]. 조사에 포함된 다른 사회적 집단 중 “받아들일 수 없다”고 응답한 비율이 동성애자보다 높았던 집단은 전과자가 69.2%로 유일했다. 북한이탈주민(12.1%)이나 외국인 이민자·노동자(7.1%), 장애인(1.3%), 결손가정자녀(1.2%)에 대한 사회적 관용도와 비교할 때, 동성애자에 대한 한국인의 일반적인 사회적 관용도는 매우 낮은 수준임을 확인할 수 있었다[34]. 또한, 2000년대 중반 이후 한국의 반동성애 운동이 빠르게 조직화되고 있으며, 성소수자를 포함한 사회적 소수자에 대한 혐오표현이 최근 중요한 사회적 문제로 부상하고 있다[35-37]. 이러한 사회적 환경에서 성소수자가 자신의 정체성으로 인해 경험하는 다양한 부정적 사회적 경험은 성소수자의 건강을 악화시키는 주요한 원인일 수 있다. 한국 연구에서도 역시 성소수자에 대한 사회적 낙인 인식과 낮은 삶의 만족도[38], 청소년 동성애자의 반동성애 폭력경험과 자살위험성[39]의 연관성이 보고된 바 있다. 한국 사회의 적대적 환경과 성소수자의 부정적 사회경험이 본 연구에서 나타난 동성애자·양성애자의 건강불평등을 얼마나 설명하는지 파악하기 위해서는 보다 다양한 후속연구가 필요하다.

본 연구 결과 중 또 다른 주목할 부분은 동성애자·양성애자 집단 내의 건강수준 차이이다. 정신건강과 신체적 증상의 경우 일반인구와 비교한 성소수자 집단의 표준화유병비가 높게 나타났지만, 자가평가건강과 건강관련행동의 경우 성소수자 집단 내에서 차이가 존재했다. 부정적 자가평가건강의 경우 여성 동성애자·양성애자 일반인구에 비해 통계적으로 유의하게 높게 나타났으며, 남성 동성애자·양성애자는 일반인구와 유의한 차이가 나타나지 않았다. 여성 동성애자·양성애자의 흡연율은 일반 여성 인구에 비해 통계적으로 유의하게 높은 것에 반해, 남성 동성애자·양성애자의 흡연율은 일반 남성 인구에 비해 통계적으로 유의하게 낮은 것으로 나타났다. 또한, 위험음주율의 경우 동성애자 여성, 양성애자 여성, 동성애자 남성 순으로 표준화유병비가 높았으며, 양성애자 남성의 경우 일반인구 유의한 차이가 나타나지 않았다.

외국의 선행연구들도 성소수자 집단 내에서 성별 및 성적지향에 따른 건강 수준의 차이를 보고하고 있으나, 각 연구 결과는 다소 다르게 나타났다[4-8]. 일부 연구는 남성에 비해 여성이 부정적

자가평가건강과 우울, 불안 등 정신건강 측면에서 높은 위험을 가진다고 보고하였고, 이성애자와 비교할 때 동성애자에 비해 양성애자 또한 비슷한 경향을 보였다[5,8,9]. 반면, 남성 동성애자 및 양성애자가 여성 동성애자·양성애자에 비해 자가평가건강 및 신체적 건강[4], 우울 및 불안 질환[13] 등 정신과적 증상 측면에서 이성애자와의 격차가 더 높게 나타난 연구결과도 존재한다. 한편, 건강관련 행동에 대한 선행연구에서는 대체로 남녀 동성애자·양성애자 모두 일반인구에 비해 높은 흡연율을 가진다는 보고가 많았으나[4,8,40], 위험음주율 또는 폭음의 경우 여성에서만 일반 인구에 비해 그 빈도가 높게 나타난 연구가 많았다[5,8,40,41].

본 연구에서 나타난 한국 동성애자·양성애자 인구집단 간 건강 수준의 차이와 선행연구의 혼재된 결과들은 성소수자 집단 내 건강 수준의 차이를 형성하는 요인과 메커니즘을 밝히기 위한 다양한 후속 연구가 필요함을 시사한다. 성소수자라 하더라도 단일한 정체성을 지니지 않으며, 성소수자 집단 내에서도 성별이나 성적지향, 연령, 사회경제적 지위 등에 따른 사회적 경험 및 건강 상 차이가 존재할 수 있다. 이러한 성소수자 집단 간 건강 수준의 차이를 형성하는 요인과 메커니즘을 밝히기 위해서는 성별이나 연령, 사회경제적 지위 등의 인구사회학적 변수들을 단순히 보정해서 분석하기 이전에 사회환경적 측면에서 면밀히 고려할 필요가 있다. 또한 한국의 경우 그동안 상대적으로 주목받지 못했던 양성애자 및 여성 동성애자·양성애자의 건강에 대한 연구가 보다 활발히 진행될 필요가 있다.

본 연구는 다음과 같은 한계를 가지고 있다. 첫째, 본 연구의 설문조사 방법과 표준화유병비 산출의 비교인구집단으로 활용된 조사의 설문 방법의 차이로 인해 표준화유병비가 과대추정되었을 가능성을 배제할 수 없다. 동일한 설문문항으로 측정하였지만 본 연구는 온라인 조사를 통해서 진행되었고, 비교대상 자료로 활용된 국가수준 건강조사는 모두 면접원을 통한 설문조사 방식으로 진행되었기 때문이다. 자살생각이나 자살시도 등 민감한 질문의 경우 면접원을 통한 설문조사에서 그 빈도가 과소보고될 가능성이 있음을 고려할 때, 이에 대한 표준화유병비가 과대추정되었을 가능성을 배제하기 어렵다. 둘째, 근골격계 통증과 건강관련행동의 경우 본 연구의 설문참여 대상은 현재 학생인 비경제활동인구를 포함하고 있는 반면, 비교대상 자료인 근로환경조사의 조사대상은 경제활동인구로 한정되어 있다. 일반적으로 경제활동 인구의 근골격계 통증과 건강위험행동의 유병률이 더 높다는 사실을 감안할 때, 본 연구의 근골격계 통증과 건강관련행동에 대한 표준화유병비는 과소추정되었을 가능성이 있다. 셋째, 본 연구의 비교집단으로 사용된 국가 대표성 있는 건강조사에는 성적지향에 대한 변수가 없었기 때문에 이들 조사에 포함된 동성애자·양성애자 참여자를 제외하고 이성애자 참여자와의 건강 수준을 비교하는 것이 가능하지 않았다. 이러한 점을 고려하여 본 연구 결과는 한국 이성애자와 동성애자·양성애자의 건강 수준을 비교한 결과가 아니라, 한국 성인 동성애자·양성애자와 이들을 포함한 한국 일반인구의 건강 수준을 비교한 결과로 제한적으로 해석될 필요가 있다.

마지막으로, 본 연구의 가장 큰 한계는 온라인 설문조사에 접근가능한 동성애자·양성애자만을 편의표집 했기 때문에 표본의 대표성을 보장할 수 없다는 것이다. 본 연구에서 설문조사 참여자의 연령대는 20-30대가 90.9%로 대다수를 차지하며, 학력 및 소득 수준도 다소 높은 편이었다. 이러한 참여자의 편중은 본 연구의 설문조사 홍보 및 참여자 모집이 기존 성소수자 단체 및 커뮤니티의 온·오프라인 연결망을 중심으로 이루어졌다는 점에서 기인했을 가능성이 있다. 저소득, 저학력, 고연령의 동성애자·양성애자들이 이러한 연결망에 대한 접근성이 낮을 수 있기 때문이다. 따라서, 연령이 높을수록 소득이나 학력 수준이 낮을수록 건강 수준이 일반적으로 낮은 경향을 고려할 때, 본 연구에서 산출된 표준화유병비는 과소추정되었을 가능성이 있다. 다만, 본 연구는 한국에서 진행된 성소수자 대상 설문조사 중 40대 이상 참여자의 비율이 가장 높았다는 점에서 의의가 있다. 본 연구는 설문조사 진행 과정에서 연령편중의 가능성을 고려하여, 성소수자 인권단체 활동가들의 인적 네트워크를 통해 40대 이상의 연구참여자들의 참여를 독려하고자 하였다. 이를 통해 본 연구의 40대 이상 참여자의 비율은 9.1%로 5% 안팎에 머물렀던 기존 한국 성소수자 설문조사[38,42]에 비해 40대 이상 연령층의 높은 참여를 이끌어 낼 수 있었다. 향후 성소수자 연구에서는 연구참여자 모집 과정에서 이러한 연구참여자의 편중을 줄이기 위한 노력이 강구될 필요가 있다.

본 연구의 한계를 넘어 동성애자·양성애자의 건강불평등 실태를 보다 체계적으로 파악하기 위해서는 국가 대표성을 지닌 연구에 참여자의 성적지향에 대한 질문이 포함되어야 한다. 북미나 유럽의 경우 성적지향을 인구통계학적 변수로 포함하는 건강조사는 점차 늘어나고 있으며, 이러한 데이터를 활용하여 성소수자의 건강불평등에 대한 다양한 연구가 활발히 진행되고 있다[4-6,9,10,12,13,43,44]. 현재 한국의 국가수준 건강조사 중 성적지향과 관련한 질문이 포함된 것은 '청소년온라인건강행태조사'가 유일하다. 이 조사는 성관계 경험의 유무에 대한 질문에 대해 이성 또는 동성과의 성관계 경험 여부로 응답하도록 하고 있다. 따라서 이를 성적 행동의 측면에서 측정된 성적지향 변수로 활용하여 동성애자·양성애자 청소년과 이성애자 청소년의 건강 수준을 비교하는 다양한 연구들이 가능해졌다[16-18]. 그동안 주목받지 못했던 성소수자 건강연구의 활성화를 위해, 향후 국민건강영양조사나 근로환경조사 등 국가수준의 건강조사에 인구사회학적 변수로 성적지향을 포함하는 것에 대한 적극적인 논의가 필요하다.

본 연구는 한국에서 진행된 가장 큰 규모로 수행된 동성애자·양성애자 대상 건강 설문조사를 이용하여, 한국 동성애자·양성

애자의 건강불평등의 정도를 파악하고자 했다. 향후 연구는 이러한 비교분석에서 한걸음 나아가 어떠한 사회적 요인이 이들의 건강을 악화시키고 또 증진시킬 수 있는지를 파악해야 한다. 더불어, 한국 성소수자의 건강불평등을 해소하기 위한 정책적 개입 방안 역시 모색되어야 한다. 외국에서는 동성애자·양성애자를 포함한 성소수자의 건강불평등 격차를 줄이기 위해 다양한 정책적 개입을 시도해왔다. 네덜란드, 스웨덴, 영국을 비롯한 미국의 다수 주에서는 차별금지법을 도입해 학교나 직장, 의료기관 등에서 성적 지향을 이유로 한 모든 차별행위를 금지하고 있다[45,46]. 또한 이와 같은 정책적 개입은 성소수자의 정신건강을 개선하는 등 성소수자의 건강불평등 격차를 해소하는 결과로 이어진 바 있다[47,48]. 본 연구에서 나타난 한국 동성애자·양성애자의 건강불평등 실태의 심각성을 고려할 때, 한국에서도 성소수자의 건강과 삶의 질을 증진하기 위해 이와 같은 정책적 개입을 도입하기 위한 노력이 적극적으로 이루어질 필요가 있다.

감사의 글

본 연구는 부분적으로 한국게이인권운동단체 친구사이와 아름다운 재단의 지원을 받아 수행되었습니다. 설문지 구성 및 설문조사 홍보 과정에서 도움을 주신 친구사이 성소수자 자살예방 프로젝트 마음연결팀 구성원 분들께 감사드립니다.

참고문헌

1. American Psychological Association. Sexual orientation and homosexuality: answers to your questions for a better understanding; 2008 [cited 2017 Jul 5]. Available from: http://www.apa.org/topics/lgbt/orientation.pdf.
2. Institude of Medicine. The health of lesbian, gay, bisexual, and transgender people: building a foundation for better understanding. Washington, DC: National Academies Press; 2011.
3. Gates GJ. LGBT demographics: comparisons among population-based surveys; 2014 [cited 2017 Jul 5]. Available from: http://williamsinstitute.law.ucla.edu/wp-content/uploads/lgbt-demogs-sep-2014.pdf.
4. Bränström R, Hatzenbuehler ML, Pachankis JE. Sexual orientation disparities in physical health: age and gender effects in a population-based study. Soc Psychiatry Psychiatr Epidemiol 2016;51: 289-301.
5. Conron KJ, Mimiaga MJ, Landers SJ. A population-based study of sexual orientation identity and gender differences in adult health. Am J Public Health 2010;100:1953-1960.
6. Sandfort TG, Bakker F, Schellevis FG, Vanwesenbeeck I. Sexual orientation and mental and physical health status: findings from a Dutch population survey. Am J Public Health 2006;96:1119-1125.
7. Sandfort TG, de Graaf R, Bijl RV, Schnabel P. Same-sex sexual behavior and psychiatric disorders: findings from the Netherlands Mental Health Survey and Incidence Study (NEMESIS). Arch Gen Psychiatry 2001;58: 85-91.
8. Dilley JA, Simmons KW, Boysun MJ, Pizacani BA, Stark MJ. Demonstrating the importance and feasibility of including sexual orientation in public health surveys: health disparities in the Pacific Northwest. Am J Public Health 2010;100:460-467.
9. Gilman SE, Cochran SD, Mays VM, Hughes M, Ostrow D, Kessler RC. Risk of psychiatric disorders among individuals reporting same-sex sexual partners in the National Comorbidity Survey. Am J Public Health 2001;91:933-939.
10. Diamant AL, Wold C. Sexual orientation and variation in physical and mental health status among women. J Womens Health (Larchmt) 2003; 12:41-49.
11. Gruskin EP, Gordon N. Gay/lesbian sexual orientation increases risk for cigarette smoking and heavy drinking among members of a large Northern California health plan. BMC Public Health 2006;6:241.
12. Steele LS, Ross LE, Dobinson C, Veldhuizen S, Tinmouth JM. Women's sexual orientation and health: results from a Canadian population-based survey. Women Health 2009;49:353-367.
13. Cochran SD, Mays VM. Relation between psychiatric syndromes and behaviorally defined sexual orientation in a sample of the US population. Am J Epidemiol 2000;151:516-523.
14. Cochran SD, Mays VM, Sullivan JG. Prevalence of mental disorders, psychological distress, and mental health services use among lesbian, gay, and bisexual adults in the United States. J Consult Clin Psychol 2003; 71:53-61.
15. Lee MH, Park JY, Kim SS. LGBTQI health research in South Korea: a systematic review. Health Soc Sci 2014;36:43-76 (Korean).
16. Yoon JH, So WY. Differences in lifestyles including physical activity according to sexual orientation among Korean adolescents. Iran J Public Health 2013;42:1347-1353.
17. Lee DY, Kim SH, Woo SY, Yoon BK, Choi D. Associations of health-risk behaviors and health cognition with sexual orientation among adolescents in school: analysis of pooled data from Korean nationwide survey from 2008 to 2012. Medicine (Baltimore) 2016;95: e3746.
18. Kim GH, Ahn HS, Kim HJ. Type of sexual intercourse experience and suicidal ideation, plans, and attempts among youths: a cross-sectional study in South Korea. BMC Public Health 2016;16:1229.
19. Jung M, Lee J, Kwon DS, Park BJ. Comparison of sexual risky factors of men who have sex with men and sex-buying men as groups vulnerable to sexually transmitted diseases. J Prev Med Public Health 2012;45:156-163.
20. Kong SU, Oh KS, No KS. The Comparison of the quality of life and mental health in homosexual men and heterosexual men. J Korean Neuropsychiatr Assoc 2002;41:930-941 (Korean).
21. Park KH. The comparison of psychopathology in homosexuality and gender identity disorder: based on candidates referred by army for medical certificate. Korean J Clin Psychol 2005;24:549-561 (Korean).
22. Jung M. Sexual behavior and condom use among gay men, female sex workers, and their customers: evidence from South Korea. PLoS One 2013;8:e66867.
23. Lee YS, Jeon CM, Kim SY, Ko BJ. Self esteem and sexual liberality of adolescent with gender identity problem or homosexual tendency. J Korean Acad Child Adolesc Psychiatry 2005;16:231-238 (Korean).
24. King M, Semlyen J, Tai SS, Killaspy H, Osborn D, Popelyuk D, et al. A systematic review of mental disorder, suicide, and deliberate self harm in lesbian, gay and bisexual people. BMC Psychiatry 2008;8:70.
25. Organization for Economic Cooperation and Development. Tackling harmful alcohol use: economics and public health policy; 2015 [cited 2017 Dec 16]. Available from: http://iogt.org/wp-content/uploads/2015/03/OECD-report-2015.pdf.
26. Organization for Economic Cooperation and Development. OECD factbook 2015-2016: economic, environmental and social statistics [cit-

ed 2017 Dec 16]. Available from: http://dx.doi.org/10.1787/factbook-2015-en.
27. Statistics Korea. Annual report on the cause of death statistics 2015; 2016 [cited 2017 Jul 5]. Available from: http://www.index.go.kr/potal/main/EachDtlPageDetail.do?idx_cd=1012 (Korean).
28. Meyer IH. Prejudice, social stress, and mental health in lesbian, gay, and bisexual populations: conceptual issues and research evidence. Psychol Bull 2003;129:674-697.
29. Kuyper L, Fokkema T. Minority stress and mental health among Dutch LGBs: examination of differences between sex and sexual orientation. J Couns Psychol 2011;58:222-233.
30. Frost DM, Lehavot K, Meyer IH. Minority stress and physical health among sexual minority individuals. J Behav Med 2015;38: 1-8.
31. Andersen JP, Zou C, Blosnich J. Multiple early victimization experiences as a pathway to explain physical health disparities among sexual minority and heterosexual individuals. Soc Sci Med 2015;133:111-119.
32. Lehavot K, Simoni JM. The impact of minority stress on mental health and substance use among sexual minority women. J Consult Clin Psychol 2011;79:159-170.
33. Roberts AL, Austin SB, Corliss HL, Vandermorris AK, Koenen KC. Pervasive trauma exposure among US sexual orientation minority adults and risk of posttraumatic stress disorder. Am J Public Health 2010;100: 2433-2441.
34. Research Center on Social Cohesion. 2015 Korea social integration survey: final report. Seoul: Korea Institute of Public Administration; 2017, p. 169-174. (Korean).
35. Yi H, Kim SS. Anti-LGBT movement in South Korea and the professionals on their side. Psychol Sex Rev 2016;7:83-85.
36. Lee NY, Baek JY. Politics of disgust: Korean conservative protestant discourses against homosexuality. J Womens Stud 2017;27: 67-108 (Korean).
37. Hong SS, Kim JH, Noh JS, Ryu MH, Lee SH, Lee JY, et al. A research on hate-speech in Korea and measures for regulation: final report. Seoul: National Human Rights Commission of Korea; 2016, p. 226-228 (Korean).
38. Kang BC. The effects of perceived social stigma on the life satisfaction of sexual minorities. Korean J Soc Welf Stud 2011;42:381-417 (Korean).
39. Kang BC, Ha KH. A study on homosexual adolescents' sexual identity and suicidal risk. Korean J Youth Stud 2005;12:267-289 (Korean).
40. Boehmer U, Miao X, Linkletter C, Clark MA. Adult health behaviors over the life course by sexual orientation. Am J Public Health 2011;102: 292-300.
41. Drabble L, Midanik LT, Trocki K. Reports of alcohol consumption and alcohol-related problems among homosexual, bisexual and heterosexual respondents: results from the 2000 National Alcohol Survey. J Stud Alcohol 2005;66:111-120.
42. Korean Society of Law and Policy on Sexual Orientation and Gender Identity. South Korean LGBTI Community Social Needs Assessment Survey. Seoul: Korean Gay Men's Human Rights Group Chingusai;2014. p. 496 (Korean).
43. Wichstrøm L, Hegna K. Sexual orientation and suicide attempt: a longitudinal study of the general Norwegian adolescent population. J Abnorm Psychol 2003;112:144-151.
44. Ward BW, Dahlhamer JM, Galinsky AM, Joestl SS. Sexual orientation and health among US adults: National Health Interview Survey, 2013. Hyattsville: National Center for Health Statistics; 2014, p. 1-12.
45. Lee JH. Disputes and perspectives on sex orientation in the legislation of anti-discrimination law in Korea. Chung-Ang Law Rev 2014;16:107-139 (Korean).
46. Jeon JY, Kim BC, Park EK, Choe YK, Soh EY. Comparative analysis on legislation and institution for gender equality [cited 2017 Aug 28]. Available from: https://www.klri.re.kr/kor/publication/pubReseachReportView.do?seq=1644 (Korean).
47. Hatzenbuehler ML, Keyes KM, Hasin DS. State-Level Policies and Psychiatric Morbidity In Lesbian, Gay, and Bisexual Populations. Am J Public Health 2009;99:2275-2281.
48. Wight RG, LeBlanc AJ, Lee Badgett M. Same-sex legal marriage and psychological well-being: findings from the California Health Interview Survey. Am J Public Health 2013;103:339-346.

부록 1. 한국 성인 동성애자·양성애자(2016)와 일반 인구(2014)[1]의 6가지 신체적 증상의 표준화유병비

			성인 동성애자·양성애자		일반 인구[1]	연령 표준화유병비	
			전체	유병률 N (%)	유병률(%)	SPR	95% CI
청력문제(N=2,322)	남성	동성애자	907	78 (8.6)	1.8	7.41	5.86, 9.25
		양성애자	121	11 (9.1)		7.96	3.97, 14.24
	여성	동성애자	617	59 (9.6)	1.1	8.44	6.42, 10.89
		양성애자	677	63 (9.3)		8.28	6.36,10.60
피부문제(N=2,325)	남성	동성애자	907	316 (34.8)	2.2	17.32	15.46, 19.33
		양성애자	120	45 (37.5)		17.56	12.81, 23.50
	여성	동성애자	618	261 (42.2)	2.7	12.75	11.25, 14.40
		양성애자	680	333 (49.0)		15.08	13.50,16.79
두통/눈의 피로(N=2,327)	남성	동성애자	909	627 (69.0)	20.1	4.56	4.21, 4.93
		양성애자	120	87 (72.5)		5.32	4.26, 6.56
	여성	동성애자	618	524 (84.8)	22.7	4.34	3.98, 4.73
		양성애자	680	604 (88.8)		4.83	4.46, 5.23
복통(N=2,321)	남성	동성애자	907	280 (30.9)	1.4	23.78	21.08, 26.74
		양성애자	120	35 (29.2)		22.78	15.87, 31.68
	여성	동성애자	617	307 (49.8)	2.0	23.27	20.74, 26.03
		양성애자	677	408 (60.3)		28.20	25.53, 31.07
호흡곤란(N=2,324)	남성	동성애자	908	77 (8.5)	0.7	17.09	13.49, 21.36
		양성애자	120	9 (7.5)		15.16	6.93, 28.77
	여성	동성애자	618	107 (17.3)	0.4	59.23	48.54, 71.57
		양성애자	678	131 (19.3)		65.32	54.62, 77.52
전신피로(N=2,326)	남성	동성애자	910	549 (60.3)	24.5	3.28	3.01, 3.56
		양성애자	121	74 (61.2)		3.64	2.86, 4.57
	여성	동성애자	618	461 (74.6)	23.6	4.24	3.87, 4.65
		양성애자	677	528 (78.0)		4.87	4.47, 5.31

SPR, standardized prevalence ratio, 표준화유병비; CI, confidence interval, 신뢰구간.
[1]제4차 근로환경조사(남성: 19-60세, 여성: 19-50세).
